# Ply Optimization of Composite Laminates for Processing-Induced Deformation and Buckling Eigenvalues Based on Improved Genetic Algorithm

**DOI:** 10.3390/ma18020345

**Published:** 2025-01-14

**Authors:** Qingchuan Liu, Xiaodong Wang, Zhidong Guan, Zengshan Li, Lingxiao Yang

**Affiliations:** School of Aeronautic Science and Engineering, Beihang University, Beijing 100191, China

**Keywords:** processing-induced deformation, finite element analysis, process simulation, genetic algorithm

## Abstract

The structure of thermoset composite laminated plates is made by stacking layers of plies with different fiber orientations. Similarly, the stiffened panel structure is assembled from components with varying ply configurations, resulting in thermal residual stresses and processing-induced deformations (PIDs) during manufacturing. To mitigate the residual stresses caused by the geometric features of corner structures and the mismatch between the stiffener-skin ply orientations, which lead to PIDs in composite-stiffened panels, this study proposes a multi-objective stacking optimization strategy based on an improved adaptive genetic algorithm (IAGA). The viscoelastic constitutive model was employed to describe the modulus variation during the curing process to ensure computational accuracy. In this study, the IAGA was proposed to optimize the ply-stacking sequence of L-shaped stiffeners in composite laminated structures. The results demonstrate a reduction in the spring-in angle to 0.12°, a 50% improvement compared to symmetric balanced stacking designs, while the buckling eigenvalues were improved by 20%. Additionally, the IAGA outperformed the traditional non-dominated sorting genetic algorithm (NSGA), achieving a threefold increase in the Pareto solution diversity under identical constraints and reducing the convergence time by 70%. These findings validate the effectiveness of asymmetric ply design and provide a robust framework for enhancing the structural performance and manufacturability of composite laminates.

## 1. Introduction

Composite materials are widely used in aerospace, automotive, and other fields due to their lightweight and high-strength characteristics. However, during the manufacturing process, composite materials inevitably generate thermal residual stresses, which affect the shape and dimensional accuracy of the structure after demolding, severely impacting its performance and causing assembly issues [1]. This problem is particularly significant for large and complex structures, where curing-induced deformation generates substantial assembly stresses, reducing the strength and fatigue life of the structure and potentially leading to structural failure [2]. The generation of curing deformation in composite laminated plates is primarily associated with temperature changes during the curing process, material anisotropy, and resin curing shrinkage. These factors interact with each other during the curing process, resulting in interlaminar residual stresses and causing complex stress fields, spring-in, and distortion deformation [3,4]. The residual stresses generated during the curing and cooling stages arise from both chemical and thermal effects [5]. Chemical shrinkage leads to resin contraction [6], while the laminate’s anisotropic mechanical properties due to the stacking sequence introduce differences in the in-plane and out-of-plane thermal expansion coefficients and chemical shrinkage coefficients of individual plies with different fiber orientations. Under the intense temperature fluctuations and complex chemical reactions during the curing process, in-plane and interlaminar residual stresses are generated, ultimately leading to curing deformation [7].

When the temperature decreases during the curing process of composite laminates, the layers will shrink in different ways due to inconsistencies in fiber orientation [8]. However, at this point, all the layers are bonded together, resulting in the generation of thermal residual stresses. For flat plate structures, the predominant deformation mode is distortion [9,10]; for structures with curvature features, such as L-shaped structures, the primary deformation mode is spring-in deformation [11]. However, for complex structures, their geometric characteristics exhibit both flat plate and curvature features. For example, a stiffened panel structure is a combination of flat plate and L-shaped structures, making the mechanism of thermal residual stresses even more complex.

To address the challenge of curing deformation in complex structures, researchers have proposed various methods, such as optimizing the curing process parameters using a manufacturer-recommended cure cycle (MRCC) [12], employing tools with thermal expansion coefficients similar to those of the prepreg material [13], and utilizing a ply design to fundamentally reduce the generation of residual stresses [1]. The utilization of a ply design is considered an effective strategy for actively controlling curing deformation [14]. By optimizing ply-stacking sequences and fiber orientations, especially through the application of asymmetric ply designs, residual stresses and deformations during the curing process can be significantly reduced, resulting in the improved overall performance of the composite material [15].

In the research and application of composite materials, ply optimization has garnered increasing attention as an effective design strategy. Ply optimization not only enhances the mechanical properties of composite materials, but also effectively addresses issues arising from curing deformation. Li employed asymmetric ply configurations to design laminates that resist hygrothermal shear deformation, achieving asymmetric ply designs for laminates comprising 8–14 layers [16]. Bruno et al. demonstrated that, by using “double–double“ layup (DD) stacking and adjusting fiber orientations, structural warping deformations can be reduced, proving that the depth of fiber orientation influences deformation [17]. Pelletier improved the strength and stiffness of materials while reducing the weight by optimizing the laminate stacking sequence and thickness [18]. Matsuzaki et al. utilized a finite element analysis and other numerical simulation methods to investigate the distribution of temperature and stress fields during the curing process, further achieving a reduction in curing deformation through an optimized ply design [19]. Cameron et al. developed a multi-objective optimization model that simultaneously optimizes the mechanical properties of materials and the deformation during the curing process, achieving more ideal design results [14].

In recent years, with the development of computer technology, the application of genetic algorithms (Gas) has gradually deepened in various fields. In the optimization of composite laminate stacking, genetic algorithms, with their strong global search capability, adaptability to discrete variables, and flexibility for multi-objective optimization, are highly effective in solving nonlinear and complex constraint optimization problems [20]. A GA is an optimization technique inspired by the principles of natural selection. As a population-based search method, it leverages the concept of “survival of the fittest” to iteratively refine solutions [21]. Some researchers [22] have applied genetic algorithms to optimize the stacking sequence of stiffened panels, improving their maximum buckling load. They have also discussed the influence of genetic parameters when using genetic algorithms to optimize stacking sequences for enhancing the buckling load. Choudhary et al. improved the buckling strength of composite cylindrical shells by 94% using genetic algorithms and a finite element analysis to optimize stacking sequences [23]. Huynh and Lee enhanced the laminate thickness design efficiency through a two-stage approach combining neural networks and genetic algorithms to predict the buckling factors [24]. Although genetic algorithms are commonly used for optimization, traditional Gas can easily become trapped in local extrema when solving more complex optimization problems [25], leading to unstable results and a low accuracy. Practical experience shows that traditional genetic algorithms often struggle to converge to a global optimum. As a result, many researchers have addressed these issues by improving the algorithm, and the introduction of the NSGA-II has greatly enhanced the stability of genetic algorithms. Srinivas and Deb [26] proposed the NSGA, a foundational method in multi-objective optimization. However, it lacks elitism, requires a sharing parameter, and has a high computational complexity. To address these issues, Deb et al. [27] introduced NSGA-II, which incorporates elitism and improves efficiency. Despite these advancements, NSGA-II struggles with diversity preservation in many-objective problems. To mitigate this, Luo et al. [28] proposed a dynamic crowding distance mechanism. Similarly, Coello and Pulido [29] developed the multi-objective micro-genetic algorithm (micro-GA), employing an archive for non-dominated solutions. Nonetheless, Pareto-based approaches often face challenges in many-objective optimization tasks [30].

Yuan et al. [31,32] proposed a multi-objective optimization method to reduce manufacturing defects and improve the efficiency in thick composite parts. Their method optimizes the curing process using Gas, combining the non-dominated sorting NSGA-II with a surrogate model, effectively reducing the curing time, the maximum temperature overshoot, and the maximum gradient of the degree of cure (DoC) simultaneously. Gao et al. [33] introduced a multi-objective genetic algorithm-based optimization strategy to address the multi-objective optimization problem in the curing process of ultra-thick composite laminates. By balancing the curing uniformity and residual stresses, their strategy achieved efficient and reliable process optimization. Fan et al. used a GA to optimize the ply layout and reduction strategy, balancing the structural weight, strength, and manufacturing performance, thereby improving the manufacturing efficiency and reliability of composite structures with ply-drop designs [34]. Kumpati et al. [35] presented a multi-objective GA for optimizing the stacking sequence of lightweight composite structures, with a focus on ensuring compliance with engineering design guidelines specific to the stacking sequence design.

The objective of this study was to solve the composite laminate stacking optimization problem through an improved adaptive GA (IAGA). First, the complex multi-field coupling processes involved in composite material manufacturing, including thermo-chemical, flow-compaction, and stress-deformation models, were analyzed, and a finite element analysis (FEA) method for the composite curing process was established. Then, based on the theoretical foundation of Gas, the role of the fitness function and genetic operators is discussed. Finally, considering the practical optimization problem—specifically, the characteristics of composite laminate stacking distributions—and the limitations of the original GA’s fixed crossover and mutation probabilities, as well as the constraints of NSGA-II, an adaptive GA was proposed. This improvement ensures a faster convergence and avoids local optima, while minimizing PIDs and residual stresses in composite components under the condition of maintaining the structural buckling stiffness, offering a more efficient and effective method for composite laminate design.

The Innovations of this paper are as follows: A high-fidelity numerical model of composite laminate PIDs, considering a simplified viscoelastic constitutive model, was established as the data input for stacking optimization, achieving a balance between data accuracy and computational efficiency. Based on theoretical derivations and the characteristics of the practical problem, an improved strategy for the GA was proposed to overcome issues such as convergence difficulties and susceptibility to local optima, which are inherent in traditional algorithms. Using an asymmetric and unbalanced ply multi-objective optimization approach, multi-objective optimization for both PIDs and the mechanical performance of the L-shaped stiffener was achieved. A characteristic index describing the influence of ply symmetry was summarized and validated with data, advancing the exploration of boundaries in composite material structure design.

In this paper, Section 2.1 introduces the relevant theories and mechanisms of PIDs, discussing the sources of PIDs, deriving the calculation of buckling eigenvalues, and providing related descriptions of the FEA. Section 2.2 analyzes the limitations of a traditional GA and proposes specific improvement strategies. In Section 3, the optimization results are analyzed and discussed, demonstrating the superior capabilities of the IAGA model. Finally, Section 4 presents the conclusion, containing clear data and summarizing the achievements of this study.

## 2. Theory and Methods

### 2.1. Curing Process Theoretical Analysis

Processing-induced deformation in composites is primarily composed of thermo-chemical, flow-compaction, and stress-deformation models.

#### 2.1.1. Thermo-Chemical Models

The temperature and cure degree distribution during the curing process of composite materials is essentially a heat conduction problem with a nonlinear internal heat source, where the internal heat source originates from the exothermic chemical reactions of the matrix resin. In this study, the mathematical model for this problem is established using Fourier’s law of heat conduction and the energy balance relationship. Based on Fourier’s law of heat conduction and the curing kinetics equation, the coupled heat conduction and resin curing dynamics lead to the heat conduction–curing analysis control equations [9,36].

(1) $$\frac{\partial }{\partial x}\left(K_{x}\frac{\partial T}{\partial x}\right)+\frac{\partial }{\partial y}\left(K_{y}\frac{\partial T}{\partial y}\right)+\frac{\partial }{\partial z}\left(K_{z}\frac{\partial T}{\partial z}\right)+\dot{q}=\rho c\frac{\partial T}{\partial t}$$

In these equations, ρ, c, T, and t represent the density, specific heat, temperature, and time, respectively; $K_{x}$, $K_{y}$, and $K_{z}$ are the thermal conductivity coefficients of the material in the global coordinates; and $\dot{q}$ is the internal heat generation rate per unit volume.

The curing kinetics part uses a phenomenological kinetic model and applies the Di-Benedetto equation to establish the relationship between the glass transition temperature and the cure degree of the QY9611 resin, as well as the glass transition temperature itself [37]. The equation is shown below, with the corresponding parameters listed in Table 1.

(2) $$\frac{d\alpha }{dt}=A\exp\left(\frac{-\Delta E}{RT}\right){\left(1-\alpha \right)}^{n}$$

(3) $$T_{g}=T_{g,0}+\frac{\left(T_{g,\infty }-T_{g,0}\right)\lambda \alpha }{1-\left(1-\lambda \right)\alpha }$$

Here, $T_{g}$ is the current glass transition temperature, $T_{g,0}$ is the initial glass transition temperature (uncured state), and $T_{g,\infty }$ is the glass transition temperature at full cure (α = 1); λ is a parameter related to the curing conditions; α is the degree of cure; and R is the universal gas constant.

#### 2.1.2. Resin Flow-Compaction Process

Based on Gutowski’s mechanistic model of resin flow/fiber interaction, prepreg laminates can be considered as a porous medium with nonlinear elastic deformation properties, filled with a viscous fluid. The excess resin that flows out of the composite material can be regarded as a seepage problem in a saturated porous medium. The flow behavior of the resin under curing pressure is described using the seepage theory of porous media. When the single-phase fluid in a porous medium is in a saturated state, assuming that the pressure during the autoclave curing process is constant, the total stress $\sigma_{ij}$ can be decomposed into two parts:

(4) $$\sigma_{ij}={\underline{\sigma }}_{ij}^{f}-\delta_{ij}P_{r}$$

where ${\underline{\sigma }}_{ij}^{f}$ represents the effective stress in the fiber; $\delta_{ij}$ is the Kronecker delta function (where $\delta_{ij}$ = 1 when i=j and $\delta_{ij}$ = 0 when i≠j and $P_{r}$ is the resin pressure).

According to Darcy’s law, the compaction equation for the composite material is given by the following:

(5) $$\frac{K_{xx}}{V_{f}}\cdot \frac{\partial^{2}P_{r}}{\partial x^{2}}+\frac{K_{yy}}{V_{f}}\cdot \frac{\partial^{2}P_{r}}{\partial y^{2}}+\frac{1}{V_{0}^{2}}\cdot \frac{\partial }{\partial z}\left(V_{f}K_{zz}\frac{\partial P_{r}}{\partial z}\right)=\mu \frac{\partial }{\partial t}\left(\frac{1-V_{f}}{V_{f}}\right)$$

**Figure 1:**
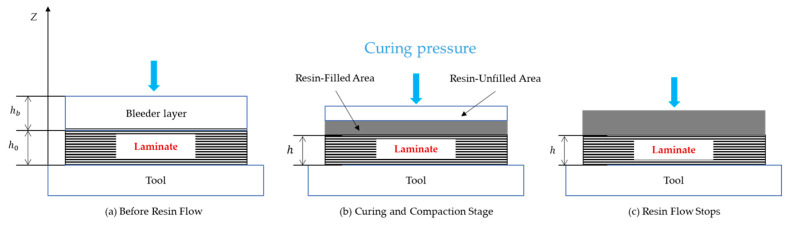
Resin flow-compaction procedure.

In the equation, $V_{0}$ represents the fiber volume fraction when the load is zero; $V_{f}$ is the fiber volume fraction; μ is the resin viscosity; and $K_{ij}$ is the permeability of the composite material. The permeability is related to the fiber volume fraction, fiber diameter, and fiber structure, with an empirical formula given by the following:

(6) $$K_{ij}=\frac{r_{f}^{2}}{4K_{0}}\cdot \frac{{\left(1-V_{f}\right)}^{3}}{V_{f}^{2}}$$

where $r_{f}$ is the fiber radius and $K_{0}$ is the Kozeny constant, which varies depending on the fiber structure and resin flow direction. According to the Kozeny–Garman theory, the material porosity is expressed as follows:

(7) $$e=\frac{Vol-V_{ol}}{Vol_{f}}=\frac{1}{V_{f}}-1$$

where Vol and $Vol_{f}$ represent the total volume of the composite material and the volume occupied by the fibers, respectively. In this study, the fiber volume fraction was set to 0.626. The process of resin flow in curing is shown in Figure 1. Figure 1 illustrates the curing process of a composite laminate under pressure. Initially, the resin is unevenly distributed with voids present. Upon applying curing pressure, the resin flows to fill the voids, creating resin-filled and unfilled areas. In the final stage, the pressure ensures complete resin penetration, resulting in a fully cured, compact, and void-free laminate.

#### 2.1.3. Stress-Deformation Process

Considering both computational accuracy and efficiency, this study used a simplified resin constitutive model similar to the viscoelastic constitutive law [38], with the following expression:

(8) $$E_{m}=\left\{\begin{array}{cc} E_{m}^{0} & T>T_{g}\left(\alpha \right)\\ {\left[\frac{T_{c}-T}{T_{c}-T_{g}\left(\alpha \right)}\right]}^{2}E_{m}^{0}+{\left[\frac{T-T_{g}\left(\alpha \right)}{T_{c}-T_{g}\left(\alpha \right)}\right]}^{2}E_{m}^{1} & T\leq T_{g}\left(\alpha \right)\;and\;T>T_{c}\\ E_{m}^{1} & T\leq T_{g}\left(\alpha \right)\;and\;T<T_{c} \end{array}\right.$$

$T_{c}$ is the characteristic temperature. Equation (8) defines $E_{m}$ as a piecewise function of the temperature T, describing the material’s modulus across different thermal regions. For $T>T_{g}(\alpha ),E_{m}$ equals the rubbery modulus $E_{m}^{0}$. For $T\leq T_{g}(\alpha )$ and $T>T_{c},E_{m}$ is a weighted combination of the rubbery modulus $E_{m}^{0}$ and glassy modulus $E_{m}^{1}$. For $T\leq T_{g}(\alpha )$ and $T\leq T_{c}$, $E_{m}$ equals the glassy modulus $E_{m}^{1}$. This captures the transition between the glassy and rubbery states.

This model differs from CHILE in that, based on the experimental results [39,40], it is assumed that $T_{g}\left(\alpha \right)$ marks the onset of modulus increase, and no parameter $T_{c1}$ is required. According to experimental data [40], the resin modulus increases rapidly at first, and then slows down as the increase completes. Therefore, the modulus does not change linearly within the ranges $T\leq T_{g}\left(\alpha \right)$ and $T>T_{c}$, but rather exponentially. Based on the fitting of the RVE model calculation results, the exponent is taken as 2. The characteristic temperature $T_{c}$ is calculated from the viscoelastic test data. The reaction kinetics and glass transition temperature of QY9611 resin are described by the following [37]:

(9) $$\frac{d\alpha }{dt}=2.28\times 10^{7}exp\left(\frac{-11114.9}{T}\right)(1-\alpha {)}^{1.485}$$

(10) $$T_{g}=-264.04+\frac{120.46\alpha }{1-0.5176\alpha }$$

**Figure 2:**
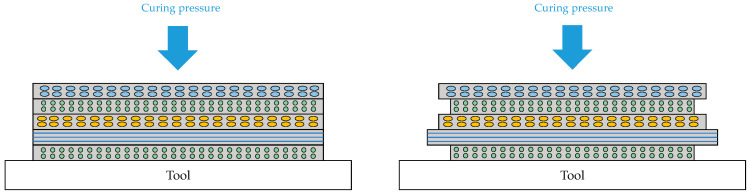
Deformation differences in ply layers with different angles.

Figure 2 illustrates the development of residual stresses during the curing process due to the mismatch in thermal and chemical shrinkage between plies with different fiber orientations. This mismatch causes interlayer residual stresses, which manifest as curing-induced deformation in the laminate.

#### 2.1.4. Calculation of Buckling Eigenvalues

The composition and stacking sequence of laminate ply ratios significantly affect the structural load-bearing performance. Given that stiffened panels exhibit a higher sensitivity to compressive loads, their mechanical performance is evaluated by calculating the buckling eigenvalues, assessing the structural stability under optimized curing deformation.

The modification of laminate stacking sequences has a significant impact on the load-bearing performance of the structure. Since stiffened panels are highly sensitive to compressive loading, their mechanical behavior is assessed by calculating the buckling eigenvalues, which serve as a crucial indicator of the structural stability.

The stiffness matrix [D] of the laminated plate is calculated based on classical laminate plate theory (CLPT):

(11) $$[D]=\sum_{k-1}^{N}\;[Q{]}^{k}\left(h_{k}-h_{k-1}\right)$$

The matrix $[Q{]}^{k}$ represents the reduced stiffness of the k-th layer, where $h_{k}$ and $h_{k-1}$ denote the positions of the top and bottom surfaces, respectively. N is the total number of layers.

Under plane stress conditions, the buckling differential equation of laminated plates is shown in Equation (12).

(12) $$D_{11}\frac{\partial^{4}w}{\partial x^{4}}+2\left(D_{12}+2D_{66}\right)\frac{\partial^{4}w}{\partial x^{2}\partial y^{2}}+D_{22}\frac{\partial^{4}w}{\partial y^{4}}+N_{x}\frac{\partial^{2}w}{\partial x^{2}}+N_{y}\frac{\partial^{2}w}{\partial y^{2}}+2N_{xy}\frac{\partial^{2}w}{\partial x\partial y}=0$$

The boundary condition is fixed at one end.

(13) $$\begin{array}{c} w(0,y)=0\\ \frac{\partial w}{\partial x}(0,y)=0 \end{array}$$

In Equation (12), w is the deflection function, $D_{ij}$ represent elements of the stiffness matrix of the laminated plate, and $N_{x},\;N_{y},\;{and\;N}_{xy}$ are the in-plane stresses.

To solve differential Equation (12), the deflection function is set as a double sine function:

(14) $$w(x,y)=Wsin\left(\frac{m\pi x}{a}\right)sin\left(\frac{n\pi y}{b}\right)$$

In Equation (14), W represents the amplitude; m,n are half-wave numbers; and a,b are the length and width of the laminated plate.

The deflection function can be substituted into the buckling differential equation to obtain the eigenvalue equation for the critical load:

(15) $$\left[D_{11}{\left(\frac{m\pi }{a}\right)}^{4}+2\left(D_{12}+2D_{66}\right){\left(\frac{m\pi }{a}\right)}^{2}{\left(\frac{n\pi }{b}\right)}^{2}+D_{22}{\left(\frac{n\pi }{b}\right)}^{4}\right]+N_{x}{\left(\frac{m\pi }{a}\right)}^{2}+N_{y}{\left(\frac{n\pi }{b}\right)}^{2}=0$$

(16) $$N_{x,cr}=\lambda_{buckling}\times N_{ref}$$

The symbols $D_{11},D_{12},D_{22}$, and $D_{66}$ represent the elements of the laminate’s bending stiffness matrix. Specifically, $D_{11}$ characterizes the bending stiffness of the plate primarily in the x-direction and $D_{22}$ does so in the y-direction, while $D_{12}$ and $D_{66}$ describe the coupling effects between different bending modes and the in-plane shear stiffness, respectively. Solving the above system of equations yields the buckling eigenvalue when $N_{ref}=1$.

#### 2.1.5. FEA Model and Mechanical Parameters

**Figure 3:**
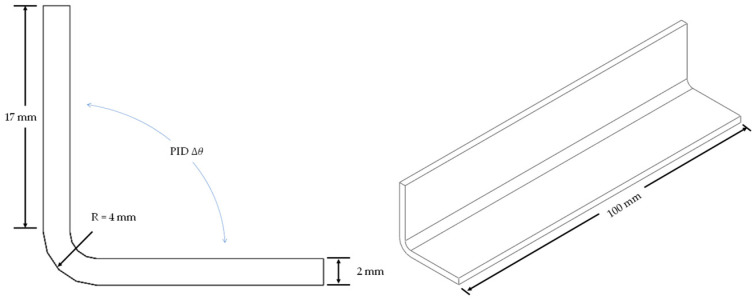
Dimensions of FEA model.

**Figure 4:**
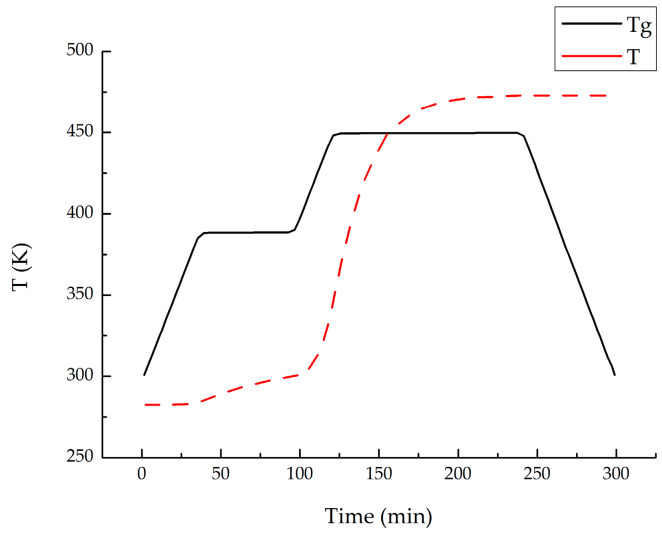
Curing process curve and degree of cure.

Considering that the primary source of deformation in stiffened panels, widely used in aircraft skin structures, is the spring-in deformation of the stiffeners causing overall structural deformations, and that stiffeners play a crucial role in bearing compressive loads [41], this study used the ABAQUS 2019 software to simulate the spring-in angle and buckling eigenvalues of a typical L-shaped laminate stiffener structure and built a GA platform using SIMULIA Isight software. Due to the limitations of shell elements in capturing the mechanical behavior in the thickness direction of the material, three-dimensional solid elements were used to model and compute the PIDs of the composite laminate [42]. The material properties were implemented through the user-defined subroutines UMAT and UEXPAN. The laminate structure was modeled using C3D20R elements, while the tool was modeled using C3D8R elements. The FEA model used in this study was validated in a previous paper published by other researchers from our research group [38]. The FEA model in this study was established with the same material parameters, boundary conditions, and loading settings, with the different dimensions as shown in Figure 3. The curing process and DoC curve are shown in Figure 4.

The mechanical properties and CTEs of ZT7H/QY9611 in a glassy state were tested, as shown in Table 2.

**Figure 5:**
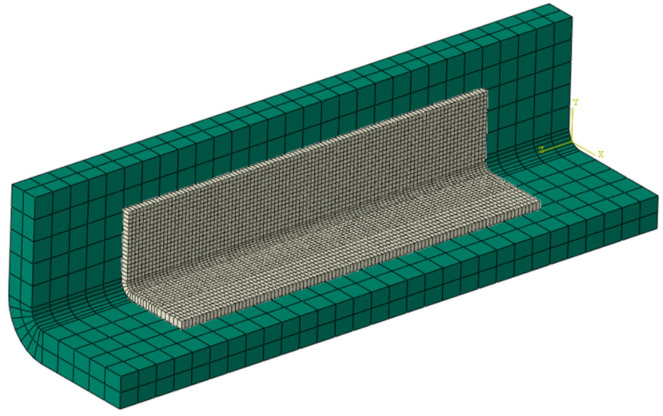
FEA model of PIDs.

**Figure 6:**
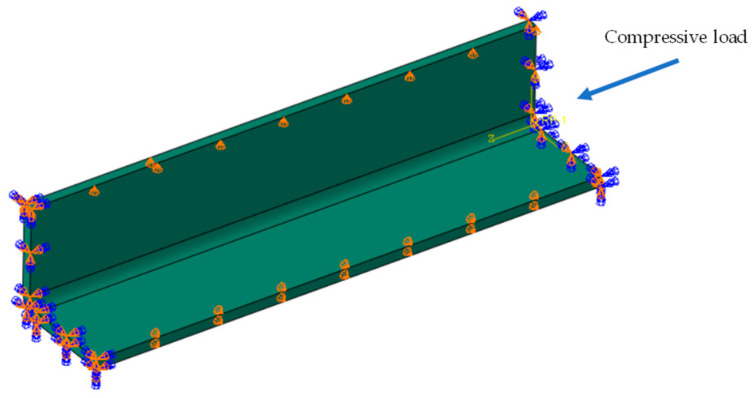
FEA model of $\lambda_{buckling}$.

The FEA models are shown in Figure 5 and Figure 6. For the FEA model of PIDs, the applied boundary conditions included the curing pressure on the surface of the L-shaped structure and frictional contact with the mold. The loading temperature curve is shown in Figure 4. After the heat preservation stage, the mold was removed using the “model change” function in Abaqus, while the L-shaped structure was fixed to allow free spring-in.

For the buckling analysis, the first-order buckling mode can clearly assess the buckling behavior of the L-shaped structure. Therefore, the buckling eigenvalue of this mode was used as the evaluation criterion. As analyzed earlier, the buckling analysis had a clear physical significance, and its accuracy was relatively higher compared to the simulation of the complex nonlinear process of curing deformation. Therefore, the trend of change in the buckling eigenvalues during the optimization process was used as the optimization objective.

### 2.2. Improved Genetic Algorithm Based on NSGA-II

The aforementioned FEA established an accurate mapping relationship between the ply angles, stacking sequences, PIDs, and buckling stiffness, providing a numerical foundation for the development of subsequent optimization models. A set of 16 plies with specific angles and stacking sequences was utilized as the inputs for the optimization model, with the PIDs and buckling stiffness defined as the optimization objectives.

#### 2.2.1. Limitations of NSGA-II in Ply Optimization

**Figure 7:**
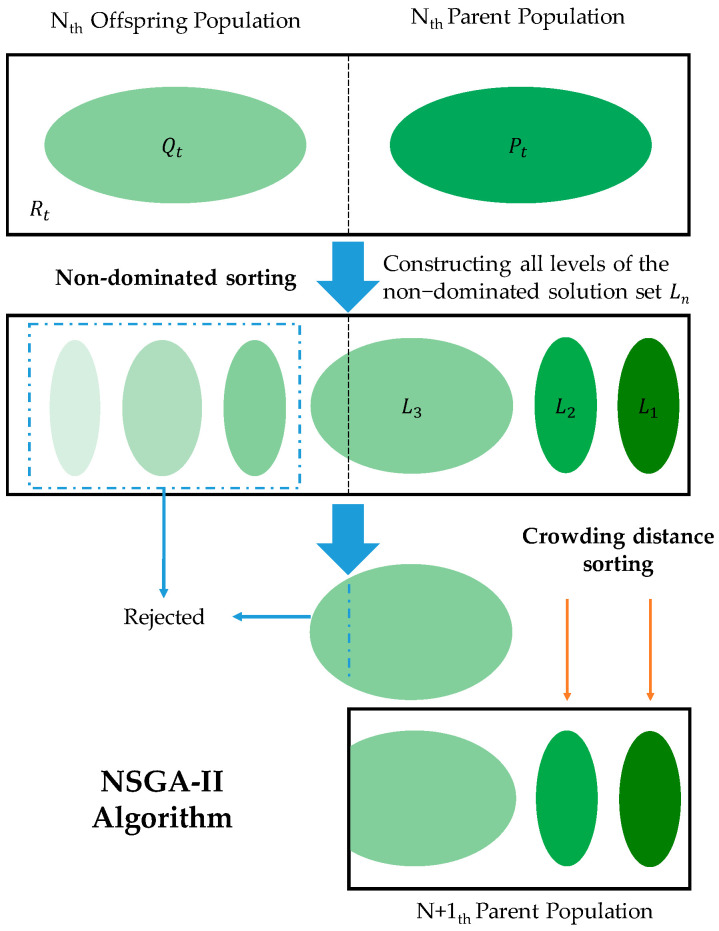
Standard NSGA-II process.

The primary distinction between the NSGA-II algorithm and a standard genetic algorithm lies in the hierarchical approach applied in NSGA-II before executing the selection operator based on the dominance relationships among individuals [43,44]. By employing a non-dominated sorting method, NSGA-II ensures that superior individuals have a higher probability of being passed on to the subsequent generation. Additionally, NSGA-II incorporates an elitism strategy, combining the parent and offspring populations to facilitate competition and produce the next generation. The standard NSGA-II process is shown in Figure 7. This figure illustrates the core workflow of NSGA-II. The parent population $P_{t}$ and offspring population $Q_{t}$ are merged into $R_{t}$ followed by non-dominated sorting to divide $R_{t}$ into ranked solution sets ($L_{1},L_{2},\ldots$). Solutions are selected based on rank and, if necessary, crowding distance to ensure diversity, forming the next-generation population $P_{t+1}$. This process balances the solution quality and diversity in multi-objective optimization. The core features are as follows: (1) the introduction of a fast non-dominated sorting algorithm; (2) the utilization of crowding distance and crowding comparison operators to maintain diversity; and (3) the implementation of an elitism strategy to preserve the best solutions.

For the stacking optimization problem addressed in this study, further modifications were made to the algorithm. These improvements mitigated the convergence challenges caused by the high input dimensionality and prevented the algorithm from becoming trapped in local optima, which can result from the highly nonlinear characteristics of a PID analysis.

#### 2.2.2. Adaptive Non-Dominated Sorting

In traditional NSGA-II, the handling of non-dominated sorting is static. However, during ply optimization, as the optimization progresses, the distribution of the population composed of 16 layers with different angles and the numerical optimization of PIDs and $\lambda_{buckling}$ continuously change. Therefore, this study introduces adaptive non-dominated sorting, dynamically adjusting the tightness of sorting based on the generational progress and changes in the objective space. This adjustment balances the maintenance of diversity and the convergence of solutions.

In the early stages, to preserve diversity, a relatively relaxed dominance relationship among solutions is allowed. In the later stages, as the solutions approach the Pareto front, the constraints on dominance relationships are gradually tightened, promoting the convergence of the solution set toward the Pareto front. This improves the algorithm’s flexibility in handling multiple ply input conditions for laminates. The factor tightness (*t*) increases progressively with the number of generations *t*, making the sorting increasingly strict:

(17) $$Tightness(t)=\frac{1}{1+exp\left(\gamma \cdot \left(t-t_{max}\right)\right)}$$

Here, γ is a constant that controls the rate of increase in tightness, and $t_{max}$ is the maximum number of generations. When t=0, the tightness⁡(t) is relatively small (approaching 0), indicating that, in the early stages, the sorting rules are more relaxed. As the number of generations increases, the tightness⁡(t) gradually grows, making the sorting rules stricter and promoting the convergence of individuals toward the Pareto solution set. The crowding distance calculation formula is modified as follows after introducing the tightness factor:

(18) $$d_{i}=\sum_{k}\;\left(\frac{f_{k}\left(x_{i+1}\right)-f_{k}\left(x_{i-1}\right)}{f_{max}(k)-f_{min}(k)}\right)\cdot Tightness(t)$$

Here, $f_{k}(x)$ represents the value of the objective function, while $f_{max}$ and $f_{max}$ are the maximum and minimum values of the objective function $f_{k}$, respectively.

#### 2.2.3. Disturbance-Driven Crossover

Traditional crossover operators tend to become conservative when handling complex nonlinear problems, making them prone to becoming trapped in local optima. By introducing a perturbation mechanism, the randomness of the crossover process is increased, maintaining the diversity of the ply population and enhancing the algorithm’s exploration capability. Additionally, the perturbation intensity decreases linearly with the number of generations to ensure convergence.

For two parent individuals $p_{1}$, $p_{2}$, and $p_{3}$, the new individual after crossover can be adjusted by incorporating perturbation as follows:

(19) $$p_{3}(i)=\left(\xi p_{1}(i)+(1-\xi )p_{2}(i)\right)+\eta \cdot N\left(0,\sigma^{2}\right)$$

Here, η is the perturbation intensity, $N\left(0,\sigma^{2}\right)$ represents standard normal distribution noise, and ξ is the crossover coefficient, ranging between 0.7 and 0.9.

#### 2.2.4. Dynamic Gaussian Mutation Operator

In multi-objective optimization problems, the algorithm needs to balance exploring the solution space (diversity) and exploiting known high-quality solutions (convergence). Traditional mutation operators, particularly those with fixed Gaussian mutation amplitudes, may lead to premature convergence (lack of diversity) or the insufficient exploration of the solution space (excessive mutation), thereby reducing the algorithm’s performance.

By replacing the mutation amplitude’s relationship with the generation *t* with an exponential decay function, the algorithm adopts a larger mutation amplitude in the early stages, which gradually decreases over time. The variation in the mutation amplitude is expressed by Equation (20).

(20) $$\sigma (t)=\sigma_{0}\cdot {\left(1-\frac{t}{T}\right)}^{\beta }$$

Here, $\sigma_{0}$ represents the initial mutation amplitude, T is the maximum number of generations, and β is the decay coefficient. As the number of generations increases, the mutation amplitude gradually decreases, reducing the disturbances to already optimized solutions.

During the optimization process, the mutation amplitude is dynamically adjusted based on the convergence status of the objectives. If a particular objective has already converged, its mutation amplitude is reduced to avoid the excessive exploration of that objective.

(21) $$\sigma_{k}(t)=\sigma_{0}\cdot {\left(1-\frac{t}{T}\right)}^{\beta }\cdot \left(1+\alpha \cdot Var\left(f_{k}\right)\right)$$

Here, Var represents the variance of objective k within the population, and α is the adjustment coefficient. The Gaussian mutation formula for generating a new solution $x^{\prime }$ is as follows:

(22) $$x_{i}^{\prime }=x_{i}+N\left(0,\sigma_{k}(t{)}^{2}\right)$$

$N\left(0,\sigma_{k}(t{)}^{2}\right)$ represents a normal distribution with a mean of 0 and a variance equal to the dynamically adjusted mutation amplitude $\sigma_{k}(t)$.

**Figure 8:**
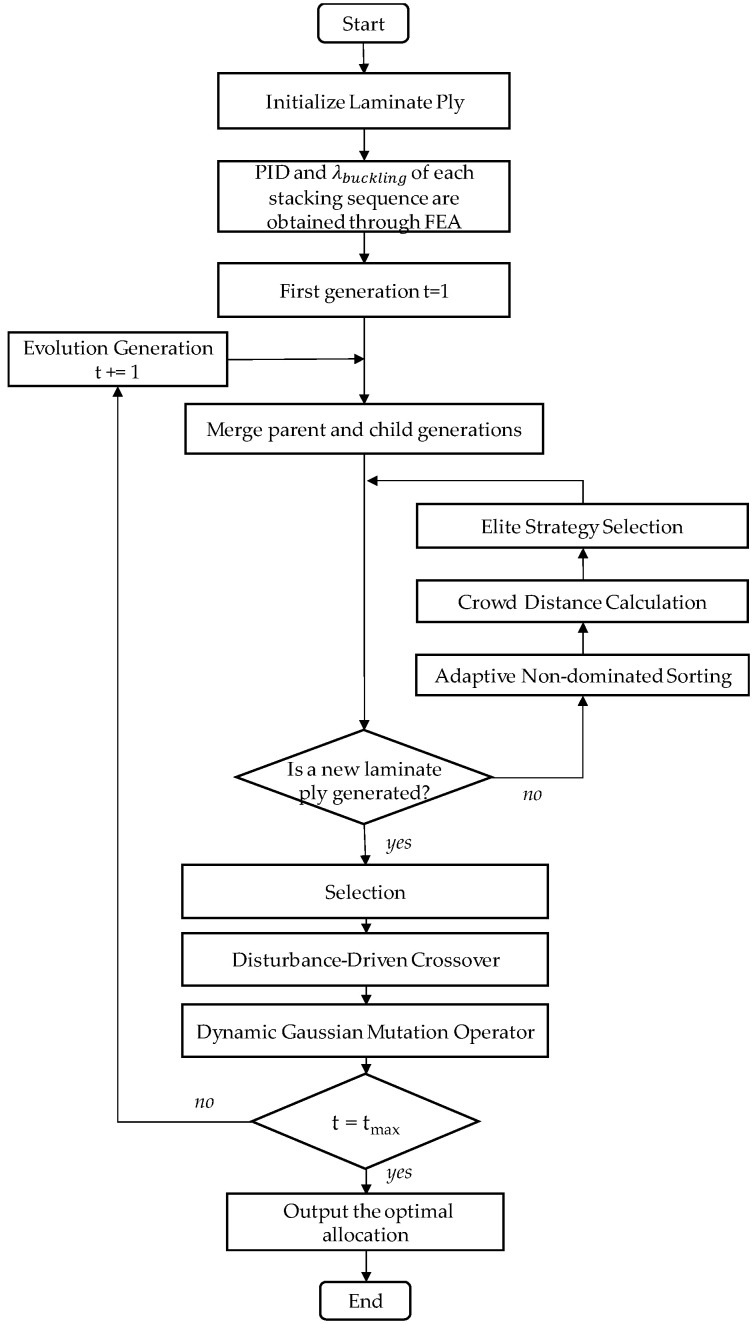
IAGA model.

The above strategy not only improves the global search capability of NSGA-II, but also enhances the local convergence speed, thereby increasing the efficiency and accuracy of the algorithm when handling complex multi-objective problems like ply optimization. The process flowchart of the IAGA model is shown in Figure 8.

#### 2.2.5. Optimization Objectives and Constraints

The stacking design constraints for laminated plates in engineering have been summarized by researchers [45]. In addition to the symmetry typically required in traditional laminate designs, the 10% rule for ply distribution must also be maintained. Specifically, for the 0°, ±45°, and 90° directions, at least 10% of the layers must be oriented in each direction.

Considering that the primary optimization objective is curing deformation, appropriate weights are assigned to focus on minimizing the curing deformation. A target aggregation method is applied to combine multiple objectives into a single comprehensive objective.

(23) $$F_{agg}(x)=\sum_{k=1}^{M}\;w_{k}f_{k}(x)$$

In this context, M denotes the total number of objectives and $w_{k}$ represents the weight assigned to the $k_{th}$ objective. We assigned a weight of 0.7 to the PID and 0.3 to $\lambda_{buckling}$.

(24) $$\left\{\begin{array}{c} Find\;X=\left(P_{1},P_{2},P_{3},\cdots ,P_{16}\right)\\ Min\{PID,\;\frac{1}{\lambda_{buckling}}\}\\ S.T.\;\text{Count}=\left({CT}_{1},{CT}_{2},{CT}_{3},{CT}_{4}\right) \end{array}\right.$$

**Figure 9:**
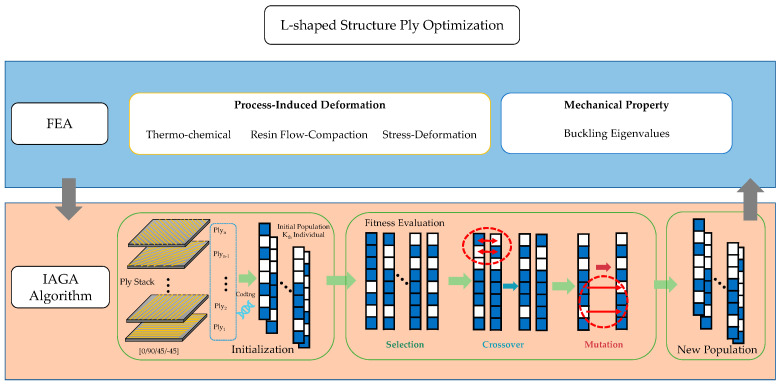
Ply optimization with FEA and IAGA.

In this constraint, P represents the ply angles, PID refers to processing-induced deformation, $\lambda_{buckling}$ denotes the maximum buckling eigenvalue, and CT indicates the statistical count of each ply type (0/±45/90). The schematic diagram of the optimization process is shown in Figure 9.

In CLPT, the *B* matrix within the *ABD* stiffness matrix characterizes the coupling between bending and tensile in composite laminates. This matrix plays a critical role in governing the spring-in deformation behavior. Consequently, this study employed the Frobenius norm of the *B* matrix as a quantitative metric to evaluate the degree of asymmetry in laminate stacking sequences, integrating it into the IAGA.

The constitutive matrix *Q* for each layer can be calculated using the uniaxial material stiffness of the composite, such as $E_{1}$, $E_{2}$, $G_{12}$, and $v_{12}$. For an isotropic material, the *Q* matrix has the following relationships:

(25) $$Q=\left[\begin{array}{ccc} \frac{E_{1}}{1-v_{12}v_{21}} & \frac{{v_{12}E}_{2}}{1-v_{12}v_{21}} & 0\\ \frac{{v_{12}E}_{2}}{1-v_{12}v_{21}} & \frac{E_{2}}{1-v_{12}v_{21}} & 0\\ 0 & 0 & G_{12} \end{array}\right]$$

where $v_{21}$ is the other component of Poisson’s ratio, which is typically calculated as follows:

(26) $$v_{21}=v_{12}\cdot \frac{E_{2}}{E_{1}}$$

In a laminate, the B matrix is the sum of the contributions of all layers to the total stiffness matrix. For each layer k, the contribution of the B matrix is as follows:

(27) $$B_{k}=Q_{k}\cdot \frac{h_{k+1}^{2}-h_{k}^{2}}{2}$$

where $h_{k}$ and $h_{k+1}$ represent the positions of the top and bottom surfaces of two adjacent layers, respectively.

For a matrix B, its Frobenius norm ${\left\|B\right\|}_{F}$ is defined as follows:

(28) $${\left\|B\right\|}_{F}=\sqrt{\sum_{i=1}^{m}\sum_{j=1}^{n}{\left|B_{ij}\right|}^{2}}$$

where $B=\left[B_{ij}\right]$ is an m×n matrix and $B_{ij}$ is the element in the i-th row and j-th column of matrix B. The relationship between ${\left\|B\right\|}_{F}$ and PID is further discussed in Appendix A.

## 3. Results and Discussion

### 3.1. Original NSGA-II Optimization Results

**Figure 10:**
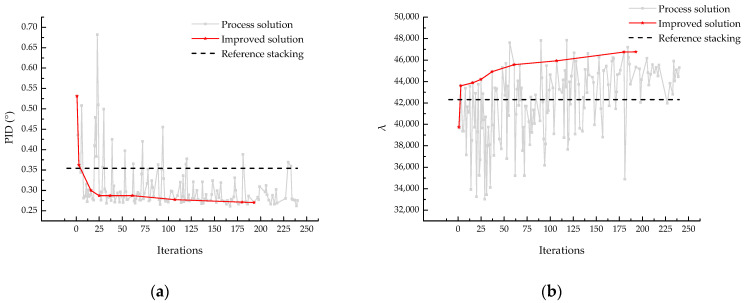
Original NSGA-II optimization curve of (**a**) PID and (**b**) $\lambda_{buckling}$.

The original NSGA-II algorithm was used to perform stacking optimization for a 16-layer L-shaped laminate. After a genetic process with a population size of 12 (*p* = 12) and 20 generations (t = 20), the optimal individual (ply stack) along with the corresponding PID and $\lambda_{buckling}$ were obtained, taking the symmetric and balanced stacking sequence [45/90/−45/0]_2s_ as a reference. The genetic curve of the optimization process is shown in Figure 10, where the horizontal axis represents the number of iterations and the vertical axis represents the optimization objective.

The gray line in the figure represents the PID and $\lambda_{buckling}$ results after each iteration using the FEA model, while the red line represents the improved solutions selected from the best individual in each generation, with the selection method also based on non-dominated sorting. Reference stacking is a commonly used symmetric balanced stacking, with a curing deformation of 0.354° and a buckling eigenvalue of 42,321. The model converged after 175 iterations, with a final curing deformation of 0.28° and a corresponding buckling eigenvalue of approximately 47,000. From the evolution curve, it can be observed that most individuals in the population tend to converge to the same extremum, while multiple near-optimal solutions are eliminated, causing the algorithm to become trapped in a local optimum. This results in an imperfect evolutionary process, which is due to the limitations of the NSGA-II algorithm in handling high-dimensional nonlinear problems.

### 3.2. Optimization Results of IAGA Model

**Figure 11:**
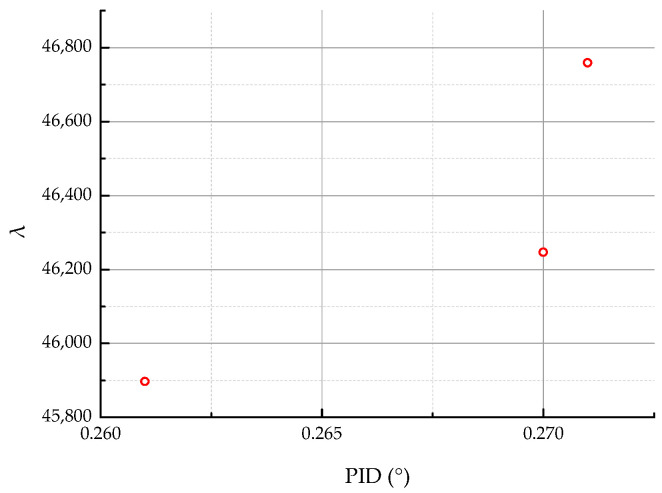
Pareto points of original NSGA-II optimization.

**Figure 12:**
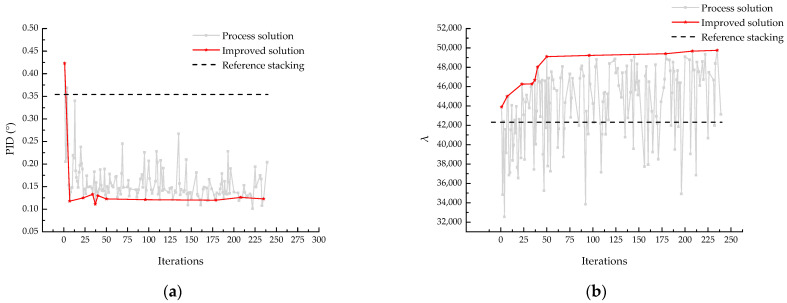
Improved model optimization curve of (**a**) PID and (**b**) $\lambda_{buckling}$.

**Figure 13:**
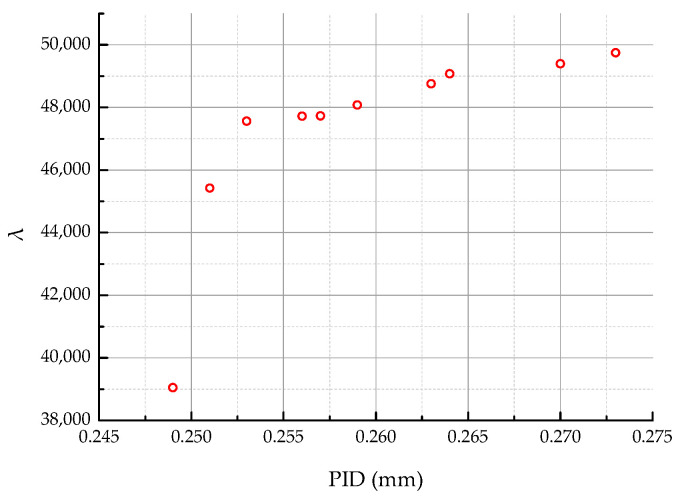
Pareto points of improved model optimization.

After 240 iterations, the current model parameters were able to identify only three Pareto points, as shown in Figure 11. This result is insufficient to meet the design requirements and does not achieve improvements in the PID. Consequently, the IAGA was developed based on the proposed strategies: adaptive non-dominated sorting, disturbance-driven crossover, and the dynamic Gaussian mutation operator. Using the same population size and genetic algorithm parameters, the results obtained are presented in Figure 12 and Figure 13.

**Figure 14:**
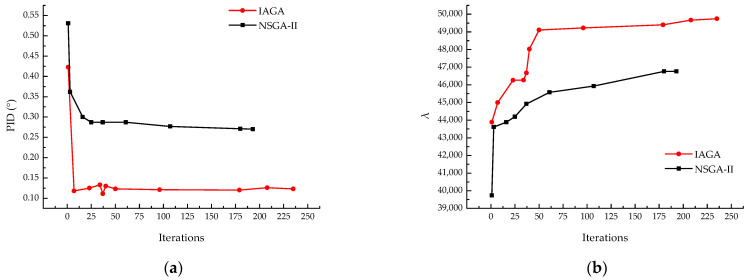
Comparison of optimization capabilities between IAGA and NSGA-II. (**a**) PID; (**b**) $\lambda_{buckling}$.

By comparing the result curves of the two models, it is evident that the IAGA proposed in this study is significantly better than NSGA-II in handling ply optimization problems. With the introduction of adaptive non-dominated sorting, the IAGA adopts a more relaxed dominance relationship between solutions, enabling it to quickly explore high-quality ply configurations with a PID value of approximately 0.12° and $\lambda_{buckling}$ of 44,000 in the initial stages. Additionally, the inclusion of a perturbation mechanism allows for the exploration of the surrounding solution space, ensuring that the identified solutions remain globally optimal rather than locally optimal. This led to the discovery of a series of ply configurations with a PID value of approximately 0.125° and $\lambda_{buckling}$ of around 50,000. The improved dynamic Gaussian mutation operator further enhanced the algorithm’s ability to explore the solution space, resulting in a greater number of Pareto points. Compared to the PID value of 0.28°, $\lambda_{buckling}$ of 46,766, and the limited number of Pareto points obtained by the traditional NSGA-II, the IAGA demonstrates not only a faster convergence, but also significantly superior optimization results, the results obtained are presented in Figure 14.

### 3.3. IAGA Incorporates Asymmetry

**Figure 15:**
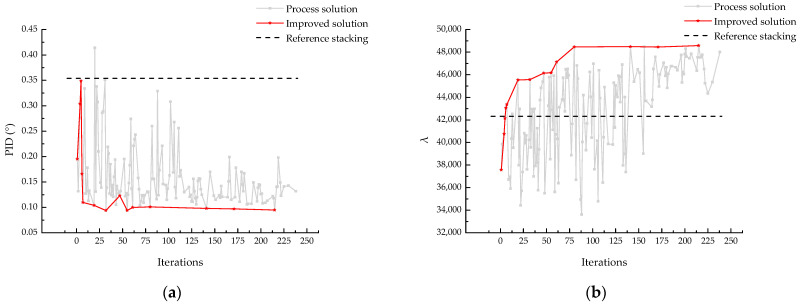
IAGA incorporates ${\left\|B\right\|}_{F}$. (**a**) PID; (**b**) $\lambda_{buckling}$.

In this study, the Frobenius norm of the *B* matrix was used as an indicator to measure the asymmetry of the laminate stacking sequence and was incorporated into the framework of the genetic algorithm for optimization. The optimization results are shown in Figure 15.

**Figure 16:**
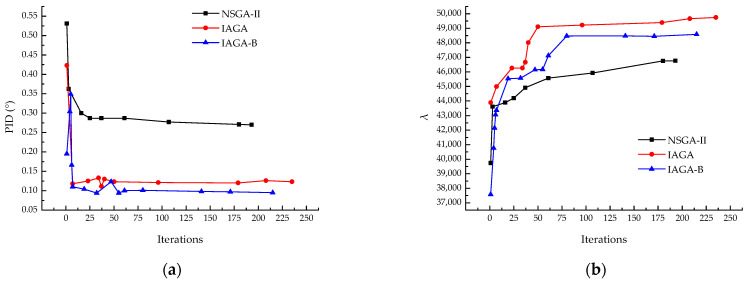
Results comparison of NSGA-II, IAGA and IAGA incorporates ${\left\|B\right\|}_{F}$. (**a**) PID; (**b**) $\lambda_{buckling}$.

**Figure 17:**
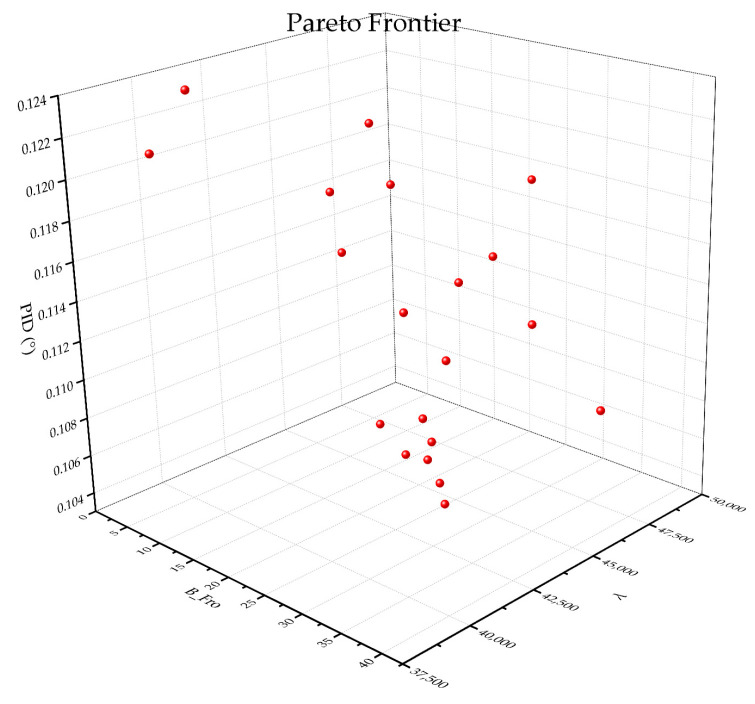
Three-dimensional Pareto front.

The results indicate that the introduction of the asymmetry indicator ${\left\|B\right\|}_{F}$ had a positive impact on the optimization problem. Compared to the model without ${\left\|B\right\|}_{F}$ in the optimization objectives, the optimization capability for PID improved by 20%, while the optimization capability for $\lambda_{buckling}$ remained consistent. However, the optimization efficiency slightly decreased, which is related to the overall complexity of the model. Figure 16 shows results of IAGA incorporates ${\left\|B\right\|}_{F}$. Figure 16 shows the result of NSGA-II, IAGA and IAGA-B. Figure 17 shows the Pareto front under the three optimization objectives.

**Figure 18:**
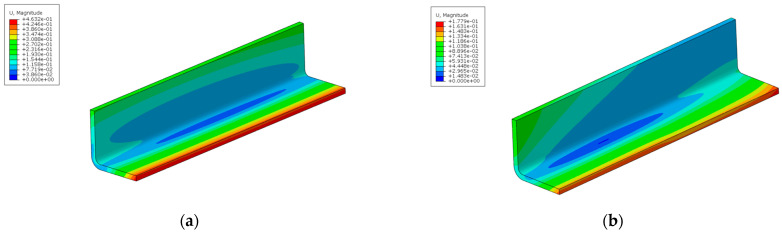
Comparison of PID before and after optimization (whole model). (**a**) Before optimization; (**b**) After optimization.

**Figure 19:**
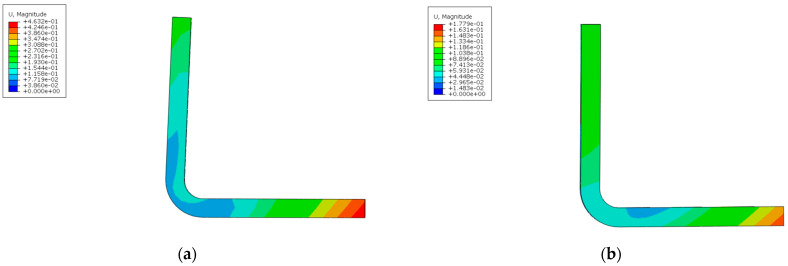
Comparison of PID before and after optimization (L-shape). (**a**) Before optimization; (**b**) After optimization.

**Figure 20:**
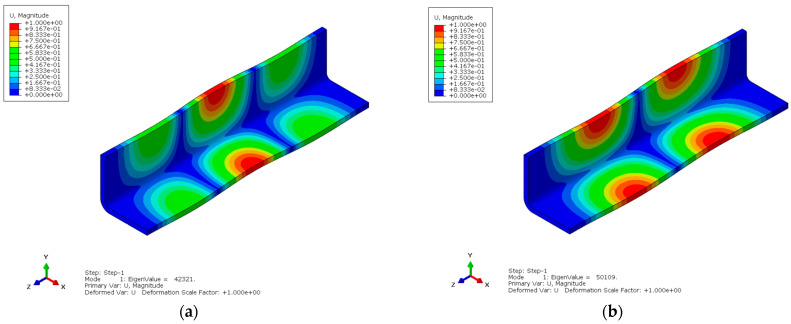
Comparison of λ before and after optimization. (**a**) Before optimization; (**b**) After optimization.

Comparing the PID and λ calculation results before and after ply optimization against the symmetric balanced stacking allows for the verification of the optimization results, as shown in Figure 18, Figure 19 and Figure 20. The deformation results after ply optimization show some differences compared to the symmetric balanced deformation pattern. The optimized results exhibit a slight twisting deformation, but it is minimal.

The buckling mode also exhibits differences, with the buckling eigenvalue of the optimized ply significantly increased.

**Figure 21:**
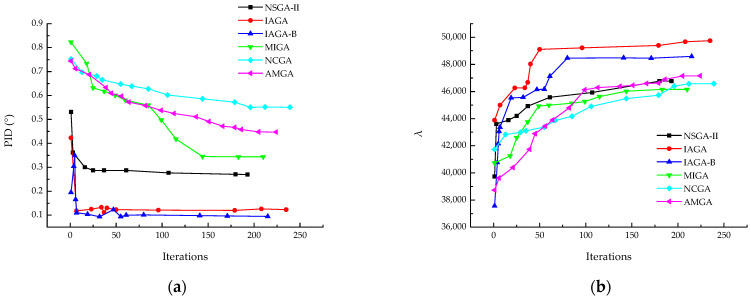
Comparison of optimization capabilities between different GA models. (**a**) PID; (**b**) $\lambda_{buckling}$.

To highlight the excellent capabilities of the IAGA algorithm for ply optimization problems, optimization calculations were performed using the multi-island genetic algorithm (MIGA), neighborhood cultivation genetic algorithm (NCGA), and archive-based micro-genetic algorithm (AMGA). The parameters for these models were set according to the default recommended settings in Isight, and the results are shown in Figure 21.

### 3.4. Influence of Population Size

**Figure 22:**
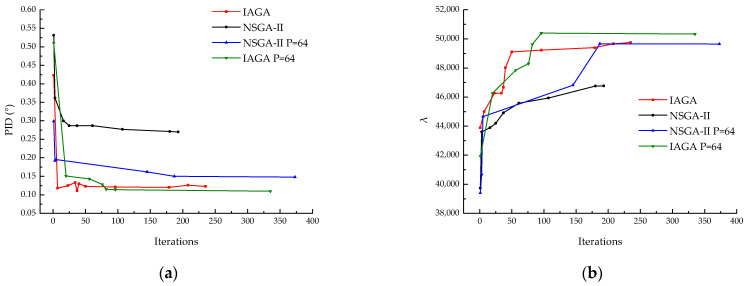
Population size of 64. (**a**) PID; (**b**) $\lambda_{buckling}$.

In this study, while using the improved NSGA-II genetic algorithm for composite ply optimization design, it was observed that the population size is a critical factor influencing the algorithm’s convergence and accuracy. Since the population size determines the number of candidate solutions in each generation, it directly affects the exploration capability of the search space and the quality of the final solution. A smaller population size reduces the computational cost, but is prone to becoming trapped in local optima, leading to a decreased robustness of the algorithm. Increasing the population size enhances the diversity, allows for broader coverage of the solution space, and provides more candidate solutions to explore the search space, thereby increasing the likelihood of finding the global optimal solution. However, a larger population size requires more fitness evaluations and selection operations in each generation, resulting in higher computational costs and the need for more computational resources and time. The comparison of computational efficiency between the two models with a population size of 64 is shown in Figure 22.

### 3.5. Computation Time

**Figure 23:**
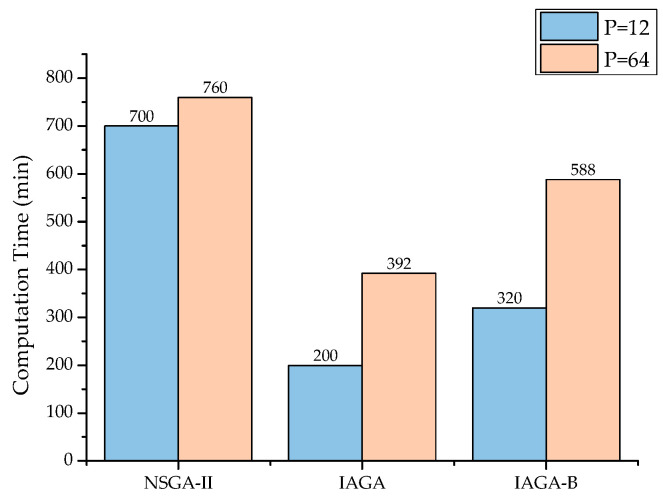
Comparison of computation times.

In industrial applications, feasible optimization solutions are inevitably constrained by their computation time and CPU requirements. Therefore, it is relevant to briefly mention the model size and computational demands. All work in this study was conducted on a workstation with Intel 12,700 K (16 logical processors) and 32 GB of RAM. The model consists of 3900 elements, with 10 CPU cores dedicated to calculating the PID and the remaining cores used for computing λ, both running concurrently. The runtime for a single iteration, including model INP file generation, solver execution, and result data transfer, was 4 min. A comparison of the computation times between the NSGA-II and IAGA models is shown in Figure 23. The computation time here refers to the total time elapsed until convergence.

The PID optimization capability refers to the percentage decrease in the optimized values of the spring-in angle and *λ* compared to the results of the reference stacking. A cross-comparison of the results shown in Table 3 from the NSGA-II, IAGA, asymmetry-enhanced IAGA-B, MIGA, NCGA, and AMGA revealed that the IAGA significantly reduced the computation time while markedly improving the optimization performance. After introducing asymmetry as an optimization objective, the convergence speed slightly decreased; however, the exploration efficiency of the model improved. This indicates that asymmetry plays an important role in PID optimization.

The improved IAGA model significantly enhanced the optimization efficiency and result quality, effectively reducing processing-induced deformations (PIDs) and improving the buckling eigenvalues. Additionally, its flexible adaptability and efficient convergence provide new insights into addressing complex multi-objective optimization problems. For laminated plates with other geometric features, based on the previous analysis, the mechanism of spring-in remains the same. Therefore, this method is equally applicable to other complex structures with corner features. However, the method relies on high-fidelity finite element analysis models, presenting limitations in terms of computational resource demands. Future research could explore the application of surrogate models, extend the approach to more complex geometries and loading conditions, and incorporate additional manufacturing constraints to enhance its practicality.

## 4. Conclusions

This study focused on multi-objective ply optimization for the PIDs and buckling eigenvalues of composite laminated structures. A high-fidelity FEA model was established, and an optimization framework was developed to address the PID of L-shaped stiffeners. Starting from the concept of asymmetric ply design, this research proposes several improvements to the traditional NSGA-II genetic algorithm, including adaptive non-dominated sorting, disturbance-driven crossover, and a dynamic Gaussian mutation operator. These proposals were derived from an in-depth analysis of the physical mechanisms underlying the curing process. The improved IAGA model significantly enhances the optimization efficiency compared to the traditional model, while also further strengthening its multi-objective optimization capabilities. It mitigates the shortcomings of the GA in handling high-dimensional nonlinear problems, such as becoming trapped in local optima, and successfully establishes an efficient and accurate improved genetic algorithm.

The improved IAGA model significantly enhances the optimization efficiency, reducing the curing deformation (PID) by 50% compared to symmetric balanced stacking, and improving the λ by 20%. Compared to the NSGA-II algorithm, the IAGA achieved a 3.5-fold improvement in convergence efficiency while delivering quantitatively superior optimization outcomes, such as identifying configurations with a PID of 0.12° and a buckling eigenvalue of over 50,000. Under the same number of iterations and constraints, the IAGA model successfully identified a Pareto solution set with a capacity of 10, representing a threefold improvement in exploration capability compared to the traditional model, which could only generate approximately three Pareto solutions. The incorporation of asymmetry as an optimization objective further enhanced the optimization capability by 20% for PIDs while maintaining the structural stability. These results validate the feasibility and advantage of applying non-symmetric ply designs in composite laminates and provide valuable insights for practical engineering applications.

Our ongoing research focuses on utilizing surrogate models to replace the FEA for PID computations, with the aim of significantly enhancing the optimization efficiency and enabling rapid ply optimization under constrained conditions. Concurrently, the feasibility of extending this methodology to accommodate more complex geometries and stacking configurations is being systematically investigated.

## Figures and Tables

**Table 1:** Curing kinetics parameters and total heat of reaction for QY9611 resin.

Material	*A*/(s^−1^)	*∆E*/(J/mol)	*n*	*H_u_*/(J/kg)	$T_{g,0}$/(K)	$T_{g,\infty }$/(K)	λ
QY9611	2.28 × 10^7^	92410	1.485	3.0032 × 10^5^	264.04	513.75	0.4824

**Table 2:** Properties of ZT7H/QY9611.

Property	*Rubbery*	*Glassy*
$E_{1}/GPa$	140	144
$E_{2}=E_{3f}/GPa$	0.16	10.2
$v_{12}=v_{13f}$	0.3	0.3
$v_{23}$	0.6	0.4
$G_{12}=G_{13f}/GPa$	0.05	6.0
$G_{23}/GPa$	0.04	3.0
$\alpha_{1}/\left(\mu \varepsilon \cdot K^{-1}\right)$	0.2	
$\alpha_{2}=\alpha_{3}/\left(\mu \varepsilon \cdot K^{-1}\right)$	40.9	
$\beta_{1}/\mu \varepsilon$	−167	
$\beta_{2}=\beta_{3}/\mu \varepsilon$	−8810	

**Table 3:** Comparison of optimization capability.

Model	Iterations to Convergence	PID Optimization Capability	λ Optimization Capability	Computation Time
NSGA-II	175	20.90%	10.50%	11.7 h
IAGA	50	64.69%	17.35%	3.3 h
IAGA-B	80	68.93%	14.80%	5.3 h
MIGA	144	2.54%	8.75%	9.6 h
NCGA	196	−55.65%	9.64%	13.1 h
AMGA	205	−26.55%	11.40%	13.7 h

**Figure A1:**
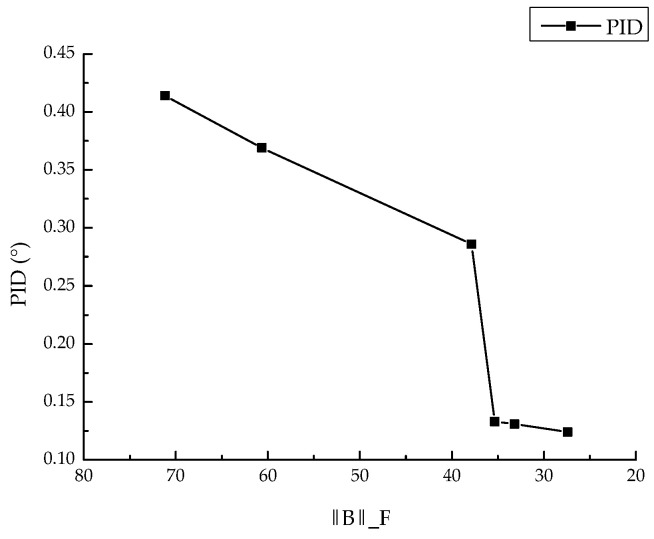
PID corresponding to ${\left\|B\right\|}_{F}$.

## Data Availability

The raw data supporting the conclusions of this article will be made available by the authors upon request.

## Nomenclature

AMGA	archive-based micro-genetic algorithm
CLPT	classical laminate plate theory
CTE	coefficient of thermal expansion
DD	“double–double” layup
DoC	degree of cure
FEA	finite element analysis
GA	genetic algorithm
IAGA	improved adaptive genetic algorithm
MIGA	multi-island genetic algorithm
MRCC	manufacturer-recommended cure cycle
NCGA	neighborhood cultivation genetic algorithm
NSGA	non-dominated sorting genetic algorithm
PID	processing-induced deformation
RVE	representative volume element
ρ	density
c	specific heat
T	temperature
t	time
$\dot{q}$	internal heat generation rate per unit volume
$K_{x}$	thermal conductivity coefficients of x-direction
$K_{y}$	thermal conductivity coefficients of y-direction
$K_{z}$	thermal conductivity coefficients of z-direction
$T_{g}$	current glass transition temperature
$T_{g,0}$	initial glass transition temperature (uncured state)
$T_{g,\infty }$	glass transition temperature at full cure (α=1)
$T_{c}$	characteristic temperature
λ	parameter of curing conditions
α	degree of cure
$\sigma_{ij}$	total stress
${\underline{\sigma }}_{ij}^{f}$	effective stress in the fiber
$\delta_{ij}$	Kronecker delta function
$P_{r}$	resin pressure
$V_{0}$	fiber volume fraction when the load is zero
$V_{f}$	fiber volume fraction
μ	resin viscosity
$K_{ij}$	permeability
$r_{f}$	fiber radius
$K_{0}$	Kozeny constant
Vol	total volume
$Vol_{f}$	volume occupied by the fibers
$E_{m}^{0}$	rubbery modulus
$E_{m}^{1}$	glassy modulus
$[Q{]}^{k}$	reduced stiffness of the k-th layer
$h_{k}$	positions of ply
N	total number of layers
$D_{ij}$	elements of the stiffness matrix of the laminated plate
$N_{x}$	in-plane stresses of x-direction
$N_{y}$	in-plane stresses of y-direction
$N_{xy}$	in-plane stresses of z-direction
γ	rate of increase in tightness
$f_{k}(x)$	value of the objective function
η	perturbation intensity
$N\left(0,\sigma^{2}\right)$	standard normal distribution noise
ξ	crossover coefficient
$\sigma_{k}(t)$	mutation amplitude
M	total number of objectives
$\lambda_{buckling}$	buckling eigenvalue
CT	statistical count
${\left\|B\right\|}_{F}$	Frobenius norm of matrix B

## Appendix A

To demonstrate the definitive influence of the ${\left\|B\right\|}_{F}$ on the PID in L-shaped structures, the PID corresponding to the ${\left\|B\right\|}_{F}$ of generated stacking sequences was calculated. The stacking sequence list is shown in Table A1, and the PID results are displayed in Figure A1.

**Table A1:** Ply sequence.

Num	Ply Sequence	${\left\|B\right\|}_{F}$	PID
P1	[45/90/-45/-45/90/-45/-45/-45/0/-45/-45/-45/0/-45/45/90]	71.17	0.414
P2	[-45/-45/45/90/-45/90/45/90/45/45/45/90/-45/90/-45/0]	33.19	0.131
P3	[0/45/45/-45/45/-45/45/90/90/45/45/90/-45/-45/45/45]	37.86	0.286
P4	[0/45/-45/90/0/-45/45/45/45/90/-45/-45/0/45/90/-45]	35.34	0.133
P5	[90/0/-45/45/-45/45/45/0/-45/45/-45/45/-45/-45/0/90]	60.64	0.369
P6	[90/45/45/0/45/45/45/90/45/0/45/0/90/45/45/-45]	27.41	0.124
